# Review of Haploidentical Hematopoietic Cell Transplantation

**DOI:** 10.1200/JGO.18.00130

**Published:** 2018-12-06

**Authors:** Mehreen A. Khan, Qaiser Bashir, Qamar-un-Nisa Chaudhry, Parvez Ahmed, Tariq M. Satti, Syed K. Mahmood

**Affiliations:** **Mehreen A. Khan**, **Qamar-un-Nisa Chaudhry**, **Tariq M. Satti**, and **Syed K. Mahmood**, Armed Forces Bone Marrow Transplant Centre/National Institute of Blood and Marrow Transplant, Rawalpindi; **Parvez Ahmed**, Quaid-e-Azam International Hospital, Islamabad, Pakistan; and **Qaiser Bashir**, MD Anderson Cancer Centre, Houston, TX.

## Abstract

Use of haploidentical (haplo) donors for hematopoietic cell transplantation (HCT) has significantly increased in the last decade. The major advantage with this strategy is universal availability and faster acquisition of the donor, along with affordability and provision of immunotherapy in post-transplantation period. Historically, haplo-HCT was associated with compromised outcomes because of high rates of graft-versus-host disease and graft failure, but after the development of a post-transplantation high-dose cyclophosphamide strategy, which results in selective T-cell depletion, these issues have been addressed to a large extent. Nevertheless, graft failure, high treatment-related mortality due to graft-versus-host disease, infections, delayed immune reconstitution, and disease relapse remain significant concerns. As the experience with haplo-HCTs grows, the clinical outcomes are becoming more at par with those seen with fully matched unrelated donor allogeneic HCTs.

## INTRODUCTION

Allogeneic hematopoietic cell transplantation (allo-HCT) is considered the only curative option for patients with various benign as well as malignant hematologic disorders.^1^ There is remarkable polymorphism in the human leukocyte antigen (HLA) gene complex, and an HLA-matched sibling donor is available in only 30% to 35% of patients.^2^ In the registries, the odds of finding a matched unrelated donor (MUD) range from 79% for white patients to 20% in minority ethnic groups.^3^ On the other hand, related donors who share at least one haplotype are available for nearly all individuals. According to an estimate, 95% of the patients have at least one haploidentical (haplo) donor, and on average there are 2.7 haplo donors available for each recipient.^4^ This universal availability makes haplo-HCT a particularly feasible option for patients who lack an otherwise fully matched donor. In this review, we summarize the historical and recent developments relating to haplo-HCT, with a particular focus on challenges in developing such a program in Pakistan.

## REVIEW OF LITERATURE

The first successful HCT was performed from an identical twin donor by Thomas et al^5^ in the late 1950s. In 1968, attempts were successful for allo-HCT from a non-twin sibling donor.^6^ The first successful unrelated donor (UD) transplantation took place in 1973. Compelled by the lack of universal availability of donors for many patients, haplo-HCT was explored.^7^ This strategy is especially helpful for ethnic minorities and resource-constrained countries where unrelated donor registries are lacking.

### Haplo-HCT With Myeloablative Conditioning Regimens

The early results of haplo-HCT were marred by the high incidence of graft failure, graft-versus-host disease (GVHD), delayed immune reconstitution, and life-threatening infections during periods of prolonged neutropenia.^8,9^ A pioneer study by Anasetti et al^10^ at the Fred Hutchinson Cancer Center showed that haplo-HCT after myeloablative (MA) conditioning is associated with increased incidence of graft failure and GVHD. This study revealed that results with a 5/6 HLA-matched donor were almost the same as with a 6/6 HLA-matched donor. However, the results with more than one antigen-mismatched graft were not encouraging. A study of 35 patients with up to three antigen-mismatched donors using MA conditioning by Powles et al^11^ revealed high incidence of GVHD (80%) with high mortality rate, thus rejecting the mismatched transplants as a feasible option.

The International Blood and Marrow Transplant Registry^12^ studied 2,000 patients including both 6/6 HLA-matched and partially mismatched donors and concluded that there is high transplantation-related mortality (TRM) associated with mismatched transplants in low/standard risk diseases, but there was a negligible difference in TRM of 6/6 HLA-matched or partially mismatched transplants in high-risk patients. Another study from Japan by the Japanese Society for HCT^13^ showed that there was a fractional difference between the outcomes of high-risk patients with fully or partially matched donor transplants, but this difference was significant in standard-risk patients.

A Japanese study by Kaida et al^14^ followed 351 patients who underwent haplo-HCT. Of those, 100 patients received MA conditioning and 251 received reduced-intensity conditioning (RIC). MA conditioning resulted in higher rates of GVHD (36%) compared with RIC (20%). Five-year overall survival (OS) was also lower with MA conditioning (30%) compared with 40% seen in patients who had received RIC (Table 1).

**Table 1:**
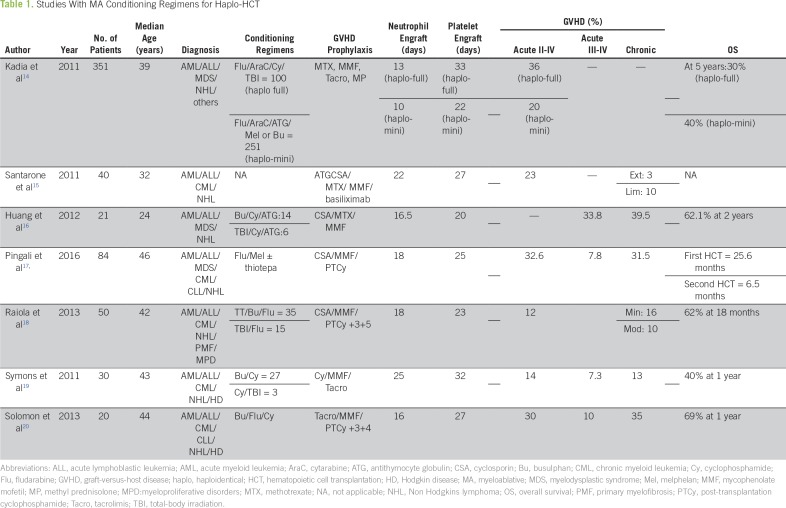
Studies With MA Conditioning Regimens for Haplo-HCT

Huang et al^16^ studied 21 patients and showed that granulocyte colony-stimulating factor (G-CSF)–primed bone marrow grafts from haploidentical donors without in vitro removal of T cells may have better results. Santarone et al^15^ also concluded that G-CSF–mobilized bone marrow harvest from haploidentical donors is a better solution for the patients who do not have fully HLA-matched donors. Many researchers demonstrated that removal of T cells from the graft attained by bone marrow harvest or peripheral blood stem cells (PBSCs) results in significantly decreased incidence of acute as well as chronic GVHD.^21^

Researchers from China^22^ compared MA conditioning regimens with antithymocyte globulin (ATG) for matched related donor (MRD) and haplo transplantations and found relatively lower incidence of acute GVHD among the partially mismatched/haplo transplantation group, probably due to in vivo T-cell depletion caused by ATG. Haplo-HCT with T-cell depletion has been reported to have high incidence of TRM, mainly as the result of infectious complications and conditioning toxicity.^23-25^ Post-transplantation cyclophosphamide (PT/Cy) has reduced TRM incidence by using T-cell–replete haplo grafts.^23^

Solomon et al^20^ studied 20 patients with myeloid malignancies who were administered MA conditioning with busulphan/fludarabine/cyclophosphamide. They observed encouraging results, with incidence of acute GVHD, chronic GVHD, and 1-year OS being 10%, 5%, and 69%, respectively (Table 1). In another report from the same group, 30 patients underwent haplo-HCT using a total-body irradiation–based MA preparative regimen. All evaluable patients achieved sustained complete donor T-cell and myeloid chimerism by day 30. The 2-year TRM was only 5%, and after a median follow-up of 17 months, the estimated 2-year OS and progression-free survival were 84% and 76%, respectively.^26^

In another report, Raiola et al^18^ studied 50 patients, with the great majority having myeloid and a few having lymphoid malignancies. Patients were administered MA conditioning, T-cell–replete grafts, and two doses of PT/Cy on day +3 and day +5 post–haplo-HCT. Incidence of acute and chronic GVHD was 12% and 10%, respectively, with relapse rate of 22% and 18% nonrelapse mortality (NRM; Table 1).

Pingali et al^17^ from MD Anderson Cancer Center followed 84 patients who received bone marrow–harvested graft after melphalan-based conditioning providing a potentially more ablative effect. Only four patients were administered PBSCs. For the patients who underwent first transplantation, median OS was 25.6 months, and the patients with second transplantation had an OS of 6.5 months. NRM was 9%, and relapse rate was 24.3%. For patients with myeloid malignancies, the results were comparable with MRD or MUD transplant, but neutrophil and platelet engraftment was longer in these patients, probably because of using bone marrow harvest as the source of stem cells.

### Nonmyeloablative Conditioning Regimens

Despite the fact that MA conditioning with megadoses of CD34+ cells (> 10 × 10^6^/kg) for haplo-HCT achieved considerable success,^27^ these regimens were not without disadvantages, including high TRM, slow engraftment/graft failure especially if megadoses of CD34+ cells were not achievable, delayed immune reconstitution, and increased GVHD mainly because of an increased number of T cells in G-CSF–mobilized grafts. Nonmyeloablative (NMA) conditioning regimens have reduced the conditioning toxicity and shortened the neutropenic period, resulting in wider application of this mode to the elderly population as well.^28^ Munchel et al^29^ at John Hopkins studied 210 patients who underwent NMA conditioning regimen for haplo-HCT followed by PT/Cy. The incidence of acute GVHD (II to IV) was 27%, chronic GVHD was 13%, and NRM was 18%, but the cumulative incidence of relapse-associated mortality was unacceptably high (55%; Table 2).

**Table 2:**
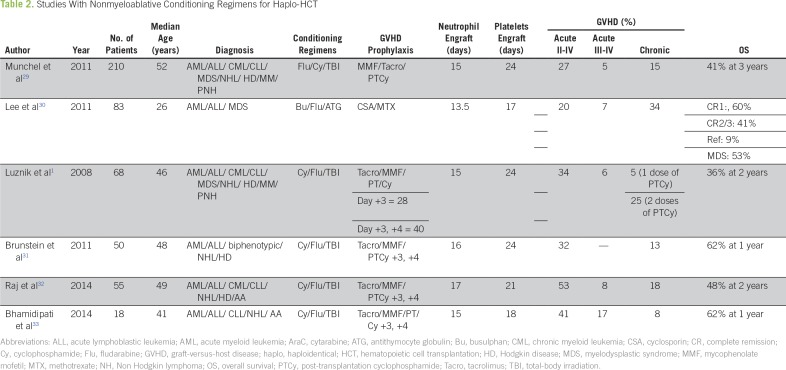
Studies With Nonmyeloablative Conditioning Regimens for Haplo-HCT

Lee et al^30^ reported the outcomes of 83 patients who underwent haplo-HCT. After a median follow-up time of 26.6 months (range, 16.8 to 78.8 months), the OS for patients with acute leukemias in first complete remission, second or third complete remission, and refractory leukemia was 60%, 41%, and 9%, respectively (Table 2).^30^

Raj et al^32^ reported the outcome of 55 patients who underwent haplo-HCT at four transplantation centers using RIC and PBSC grafts followed by PT/Cy. The incidence of acute GVHD grade II, III, IV, and chronic GVHD was 53%, 8%, 0%, and 18%, respectively. OS was 48% at a median follow-up of 509 days (Table 2).

Luznik et al^1^ and Bhamidipati et al^33^ also used an NMA conditioning regimen for their patients undergoing haplo-HCT. Luznik et al^1^ reported 50% OS at 4.1 years median follow-up, and Bhamidipati et al^33^ reported 62% OS at 1 year. Significant clinical studies with MA/NMA conditioning revealed results comparable to matched sibling donor (MSD)/MRD/MUD transplants, but additional validation of the data is awaited.^34-86^

## HAPLOIDENTICAL STEM-CELL TRANSPLANTATION: PAKISTANI PERSPECTIVE

Pakistan is the sixth most populous country, with more than 200 million inhabitants. The country is facing many challenges, including population explosion, illiteracy, lack of nationwide health infrastructure, economic crises, and, last but not the least, war on terrorism.

The incidence of aplastic anemia (AA) in Asia is two- to three-fold higher than the incidence in any other part of the world.^87^ In Pakistan, more than 50% of patients in any hematology clinic have a diagnosis of AA.^88^ Etiology of the high incidence of AA includes both genetic and acquired factors.^89^ Host genetic factors, especially short telomere length, have been recognized to be one of the culprits. Environmental factors responsible for increased incidence of AA include pesticides, benzene, arsenic, and many viruses. In Pakistan, the rural population is at a greater risk of AA compared with the urban population, because, being an agricultural country, people living in villages have increased exposure to pesticides.^90^ Pesticides have been reported to be associated with hematologic malignancies as well. Exposure to excessive radiation also increases the risk of acute and chronic leukemia.

Among genetic disorders, the most frequently encountered hemoglobinopathy is β-thalassemia major. There are more than 100,000 patients with β-thalassemia major in the country, and, despite increasing awareness about prevention, 5,000 annual births are still being reported.^91^ HCT is the only curative option for AA, β-thalassemia major, and poor-risk leukemias.

## CURRENT STATUS OF HCT IN PAKISTAN

In Pakistan, only eight well-established centers are carrying out hemopoietic stem-cell transplantation, and few smaller centers are also being developed. The first bone marrow transplantation center was established in 1999 in Karachi (National Institute of Blood Disease) followed by Armed Forces Bone Marrow Transplant Centre in 2001, which has emerged as the largest center of the country. Aga Khan University Hospital was started in 2004.

**Fig 1:**
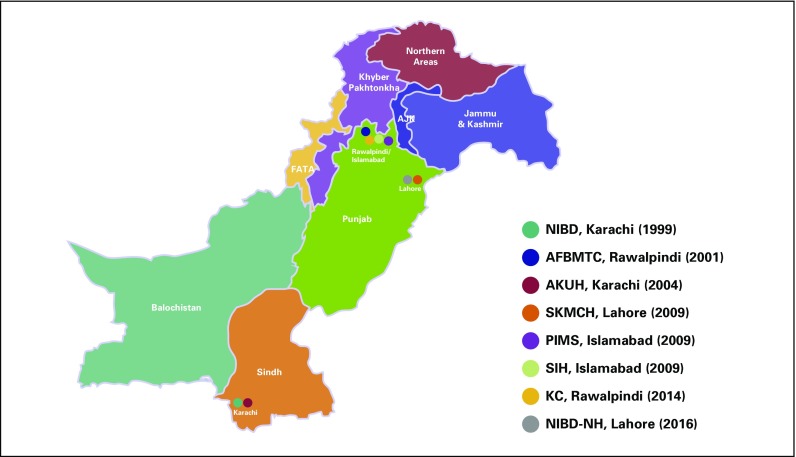
Transplantation centers in Pakistan. NIBD, National Institute of Blood Disease; AFBMTC/NIBMT, Armed Forces Bone Marrow Transplant Centre/National Institute of Blood and Marrow Transplant; AKUH, Aga Khan University Hospital; SKMCH & RC, Shaukat Khanam Memorial Cancer Hospital and Research Centre; PIMS, Pakistan Institute of Medical Science; SIH, Shifa International Hospital; KC, Kidney Centre; NIBD-NH, National Institute of Blood Disease–National Hospital.

A total of 1,589 transplantations had been carried out by 2017 (data collected by personal communication), out of which 256 were autologous. The major indications for transplantation were β-thalassemia major (603), followed by aplastic anemia (540) and acute leukemia (169). The patient population comprised both children and adults (Fig 1).

All patients received stem-cell grafts from matched related donors. However, these transplantations account for a small percentage of the potential candidates requiring HCT in our country.

## MAJOR INDICATIONS FOR HCT IN PAKISTAN

Major indications for HCT in our country are listed in Table 3. Most of the patients being transplanted belong to the groups of mentioned diseases, but few very rare diseases have also undergone HCT.

**Table 3:**
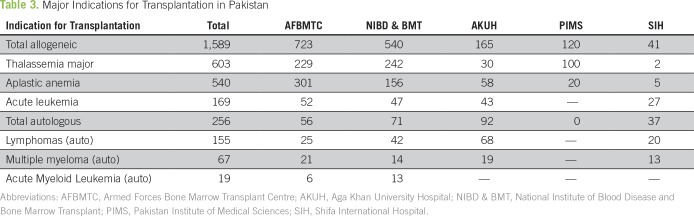
Major Indications for Transplantation in Pakistan

## HAPLO-HCT IN PAKISTAN

**Fig 2:**
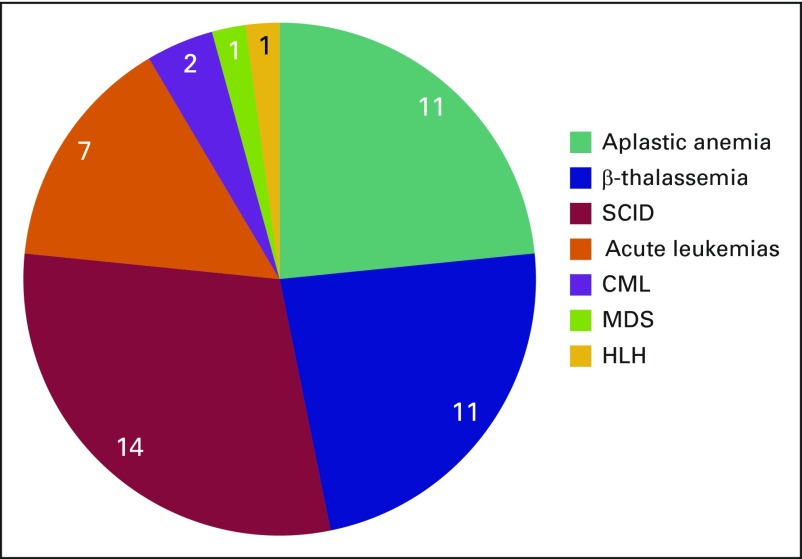
Major indications for haploidentical transplantation in Pakistan. CML, chronic myeloid leukemia; HLH, haemophagocytic lymphohistiocytosis; MDS, myelodysplastic syndrome; SCID, severe combined immunodeficiency diseases.

Currently only two larger transplantation centers of Pakistan are carrying out haplo-HCT. A total of 47 haplo-HCT procedures have been performed in the country for various indications, including aplastic anemia, β-thalassemia major, immunodeficiency states, and acute leukemias (Fig 2).

## CURRENT CHALLENGES FOR HCT IN PAKISTAN

Less than 10% of the potential candidates requiring HCT can actually undergo the procedure for the following reasons:

1. Lack of specialized centers.
2. Lack of trained human resources, including doctors, nurses, pharmacists, technologists, and paramedics. Low HCT team density: there are just five HCT teams for a population of 200 million (ie, 0.025 teams/million), compared with Europe, where the ratio is 14.43/million, and Eastern Mediterranean countries, with a ratio of 1.55/million population.^92^
3. Limited availability of specialized instruments.
4. High cost involved in the procedure.
5. Limited availability of fully HLA-matched donors.
6. Nonavailability of MUD registry and cord blood banks.
7. Extremely high cost required for approaching international MUD registries and cord blood banks.
8. Lack of infrastructure and government support for the national transplant registry.

Because of these constraints, haplo-HCT seems to be the most feasible transplantation option for patients who do not have a fully HLA-matched donor.

## CONCLUSION AND FUTURE DIRECTIONS

Haplo-HCT seems to be a feasible way to provide transplantation for patients who need allo-HCT but lack a fully HLA-matched donor. This seems particularly true for the developing countries, where lack of resources poses a major challenge. The use of high-dose cyclophosphamide day +3 and +4 post–haplo-HCT is an effective strategy to deplete alloreactive T cells responsible for GVHD and graft rejection. This does not harm stem cells in the graft, which are resistant to cyclophosphamide because of high levels of the enzyme aldehyde dehydrogenase. Ongoing studies comparing haplo-HCT with cord blood transplantations or MUD transplantations will shed light on preference of haplo donors if an MRD is not available.
